# The association between adiposity and anti-proliferative response to neoadjuvant endocrine therapy with letrozole in post-menopausal patients with estrogen receptor positive breast cancer

**DOI:** 10.1038/s41523-022-00453-7

**Published:** 2022-08-04

**Authors:** Edoardo Isnaldi, François Richard, Maxim De Schepper, Sophia Leduc, Marion Maetens, Tatjana Geukens, Karen Van Baelen, Ha-Linh Nguyen, Ghizlane Rouas, Gabriele Zoppoli, Fatima Cardoso, Christos Sotiriou, Denis Larsimont, Giuseppe Floris, Elia Biganzoli, Christine Desmedt

**Affiliations:** 1 KU Leuven, Department of Oncology, Laboratory for Translational Breast Cancer Research, B-3000 Leuven, Belgium; 2 Department of Internal Medicine and Medical Specialties, University of Genoa, IT-16132 Genoa, Italy; 3 University Hospitals Leuven, Department of Pathology, B-3000 Leuven, Belgium; 4 Université Libre de Bruxelles, Institut Jules Bordet, J.C. Heuson Breast Cancer Translational Research Laboratory, B-1000 Brussels, Belgium; 5 Ospedale Policlinico San Martino IRCCS per l’Oncologia, IT-16132 Genoa, Italy; 6 Champalimaud Clinical Center-Champalimaud Foundation, Breast Unit, P-1400-038 Lisbon, Portugal; 7 Université Libre de Bruxelles, Institut Jules Bordet, Pathology Department, B-1000 Brussels, Belgium; 8 KU Leuven, Translational Cell and Tissue Research Unit, Department of Imaging and Pathology, B-3000 Leuven, Belgium; 9 Unit of Medical Statistics, Biometry and Epidemiology, Department of Biomedical and Clinical Sciences (DIBIC) “L. Sacco” & DSRC, LITA Vialba campus, Università degli Studi di Milano, IT-20157 Milan, Italy

**Keywords:** Breast cancer, Tumour biomarkers

## Abstract

The impact of adiposity on the efficacy of endocrine treatment in patients with estrogen receptor positive breast cancer is poorly investigated. Here, we retrospectively investigated in a cohort of 56 patients whether body mass index and/or mammary adiposity are associated with anti-proliferative response in the neoadjuvant setting. Anti-proliferative response was defined as high Ki67 at baseline (Ki67_bl_) and low Ki67 at surgery (Ki67_srg_), using the 14% cut-off. Mammary adipocyte size was assessed on hematoxylin and eosin slides from the surgical samples using digital pathology. A higher proportion of tumors with an anti-proliferative response was observed in patients with obesity (54.5%) as compared to patients with normal weight (9.0%) and patients with overweight (40.0%) (*p* = 0.031), confirmed by multivariable regression analysis adjusted for baseline Ki67 (OR, obese vs normal weight: 13.76, 95%CI: 1.49–207.63, *p* = 0.020). Larger adipocyte diameter was identified as predictor of anti-proliferative response (OR per increase in diameter of 5 μm for adipocytes distant from the tumor: 2.24, 95%CI: 1.01–14.32, *p* = 0.046). This study suggests that anti-proliferative response to neoadjuvant letrozole might be more frequent in patients with increased systemic or mammary adiposity.

## Introduction

The overweight and obese post-menopausal female population is exposed to an increased risk of developing breast cancer (BC) that it is tightly linked to the estrogen receptor (ER)-positive subtype^1^. Increased adiposity, standardly defined by an elevated body mass index (BMI), has prognostic implications, leading to worse outcomes across all BC molecular subtypes^2^. Also, it might affect response to various BC treatments including endocrine treatment (ET), although the underlying mechanisms are mostly still unknown^3–5^.

While BMI is a commonly used measure of individual’s adiposity, it does not accurately describe the fat mass located in different adipose depots^6^. Recently, the adipocyte size has been used as alternative measure of adiposity, giving information on adiposity in specific body sites such as the mammary adipose tissue^7^. The mammary adipose tissue of women with overweight, obesity and of a non-negligible percentage with normal weight can exhibit a low-grade inflammatory state characterized by adipocyte hypertrophy, macrophage infiltration with the formation of crown-like structures (CLS), and it is associated with a series of changes in the breast adipose tissue microenvironment, including a local increase of aromatase activity^8,9^. During BC initiation and growth, there is a remodeling of the mammary adipose tissue in which cancer cells engage in close contact with adipocytes determining the conversion of adipocytes into the so-called cancer-associated adipocytes (CAAs). CAAs take an active part in the tumor microenvironment, releasing free-fatty acids, inflammatory cytokines, adipokines and other molecules to support the tumor growth^10^.

Most of the patients with ER-positive BC receive ET after surgery^11^. Recently, several neoadjuvant clinical trials have demonstrated that neoadjuvant ET achieves surgical outcomes similar to neoadjuvant chemotherapy with the advantage of less toxicity^12–14^. Several trials defined changes in Ki67, measured on pre-treatment and “on-treatment” tumor specimens, as an early biomarker of anti-proliferative response, associated with long-term prognosis^15–17^. While inconclusive results have been reported regarding the differential efficacy of ET (aromatase inhibitors-AIs versus tamoxifen), according to patient’s BMI in the adjuvant setting^18^, to our knowledge only two studies evaluated the efficacy of AIs in the neoadjuvant setting according to patient’s BMI^19,20^. Takada et al. found that the efficacy of exemestane, measured as clinical response, was greater in patients with high BMI compared to patients with low BMI^19^. In a more recent study, Franzoi et al. showed no significant difference in Ki67 changes and radiological response according to BMI in patients receiving neoadjuvant anastrozole plus abemaciclib^20^. Thus far, no clinical study has investigated the relationship between obesity/mammary adiposity and anti-proliferative response following neoadjuvant letrozole.

In the present study, we retrospectively evaluated whether BMI and the size of the mammary adipocytes are associated with the anti-proliferative response to neoadjuvant aromatase inhibition with letrozole. We further explored whether mammary adiposity was associated with the presence of CLS and tumor-associated macrophages (TAM) and whether mammary adiposity differs according to the main BC histological subtypes (invasive lobular carcinoma—ILC versus invasive carcinoma of no special type –NST, formerly called ductal carcinoma).

## Results

### Patient characteristics and change in Ki67

In the present study, we considered 56 out of 66 patients that were treated at the Institut Jules Bordet with neoadjuvant letrozole in the context of the Fragrance trial (NCT00199134) (supplementary Fig. 1). Pre- and post-treatment patient and tumor characteristics are summarized in Table 1 according to BMI category. BMI did not show any evidence of association with the standard clinico-pathological characteristics. 13 (23.2%), 20 (35.7%), and 23 (41.1%) out of the 56 patients were normal weight, overweight, and obese, respectively. Distant adipocytes and CAAs were digitally measured in 39 out of 56 (69.6%) and 33 out of 56 (58.9%) patients, respectively. The lower percentage of samples examined for CAAs is justified by the fact that the regions close to the tumor presented globally a minor quality due to fibrosis and damaged adipose tissue. We observed a conversion of ER status from positive to negative from baseline to surgery in 2/56 (3.5%) whereas we observed a negative conversion of progesterone receptor (PgR) in 17/46 patients (36.9%). It can be explained by the effect of letrozole that has been reported to significantly reduce the expression of the estrogen-regulated protein PgR^21^. In Supplementary Table 1 we reported the Quick Score for ER and PgR at baseline and at surgery.

**Table 1:** Patient and tumor characteristics according to BMI category.

Patient and tumor characteristics	All	Normal weight	Overweight	Obese	*P*-value
	(*n* = 56)	(*n* = 13)	(*n* = 20)	(*n* = 23)	
Age (continuous), years					0.667
Mean (SD)	68 (8.0)	67.6 (7.6)	69.4 (7.3)	67.0 (9.0)	
Median (IQR)	68.5 (62–74)	69 (64–71)	70 (63.8–74)	67 (59.5–74.5)	
Range	51–83	54–80	58–83	51–82	
Age, years					0.819
<65	19 (33.9)	4 (30.8)	6 (30.0)	9 (39.1)	
≥65	37 (66.1)	9 (69.2)	14 (70.0)	14 (60.9)	
Tumor grade (baseline)					0.364
G1	9 (17.0)	2 (16.7)	5 (26.3)	2 (9.1)	
G2	39 (73.6)	10 (83.3)	13 (68.4)	16 (72.7)	
G3	5 (9.4)	0 (0.0)	1 (5.3)	4 (18.2)	
Missing	3	1	1	1	
Tumor grade (surgery)					0.438
G1	19 (33.9)	2 (15.4)	8 (40.0)	9 (39.1)	
G2	32 (57.1)	10 (76.9)	11 (55.0)	11 (47.8)	
G3	5 (9.0)	1 (7.7)	1 (5.0)	3 (13.1)	
Tumor size (cT), cm					0.620
≤2	3 (5.5)	0 (0.0)	2 (10.0)	1 (4.6)	
2.1–5	42 (76.3)	12 (92.3)	14 (70.0)	16 (72.7)	
>5	10 (18.2)	1 (7.7)	4 (20.0)	5 (22.7)	
Missing	1	0	0	1	
Tumor size (pT), cm					0.218
≤2	22 (39.3)	7 (53.8)	9 (45.0)	6 (26.1)	
2.1–5	27 (48.2)	6 (46.2)	7 (35.0)	14 (60.9)	
5	7 (12.5)	0 (0.0)	4 (20.0)	3 (13.0)	
ER status (baseline)					1
Negative	0 (0.0)	0 (0.0)	0 (0.0)	0 (0.0)	
Positive	56 (100)	13 (100)	20 (100)	23 (100)	
ER status (surgery)					0.709
Negative	2 (3.6)	1 (7.7)	0 (0.0)	1 (4.3)	
Positive	54 (96.4)	12 (92.3)	20 (100)	22 (95.7)	
PgR status (baseline)					0.744
Negative	10 (17.8)	3 (23.1)	4 (20.0)	3 (13.0)	
Positive	46 (82.2)	10 (76.9)	16 (80.0)	20 (87.0)	
PgR status (surgery)					1
Negative	26 (47.3)	6 (46.2)	10 (50.0)	10 (45.5)	
Positive	29 (52.7)	7 (53.8)	10 (50.0)	12 (54.5)	
Missing	1	0	0	1	
HER-2 status (baseline)					1
Negative	54 (98.2)	12 (100)	20 (100)	22 (95.7)	
Positive	1 (1.8)	0 (0.0)	0 (0.0)	1 (4.3)	
Missing	1	1	0	0	
HER-2 status (surgery)					0.825
Negative	51 (92.7)	11 (91.7)	18 (90.0)	22 (95.7)	
Positive	4 (7.3)	1 (8.3)	2 (10.0)	1 (4.3)	
Missing	1	1	0	0	
Ki67 % (baseline)					0.432
0–13	26 (48.1)	7 (63.6)	10 (50.0)	9 (39.1)	
≥14	28 (51.9)	4 (36.4)	10 (50.0)	14 (60.9)	
Missing	2	2	0	0	
Ki67 % (surgery)					0.491
0–13	47 (87.0)	9 (75.0)	18 (90.0)	20 (90.9)	
≥14	7 (13.0)	3 (25.0)	2 (10.0)	2 (9.1)	
Missing	2	1	0	1	
Type of surgery					0.385
Breast-conservative surgery	36 (64.3)	7 (53.8)	15 (75.0)	14 (60.9)	
Mastectomy	20 (35.7)	6 (46.2)	5 (25.0)	9 (39.1)	
Histology (surgery)					0.160
NST	38 (67.9)	8 (61.5)	11 (55.0)	19 (82.6)	
ILC	18 (32.1)	5 (38.5)	9 (45.0)	4 (17.4)	
Nodal status (surgery)					0.821
0	30 (53.6)	8 (61.5)	11 (55.0)	11 (47.8)	
1–3	19 (33.9)	3 (23.1)	7 (35.0)	9 (39.1)	
≥4	7 (12.5)	2 (15.4)	2 (10.0)	3 (13.1)	
PEPI score					0.303
0	1 (1.9)	1 (8.3)	0 (0.0)	0 (0.0)	
1–3	29 (53.7)	8 (66.7)	10 (50.0)	11 (50.0)	
≥4	24 (44.4)	3 (25.0)	10 (50.0)	11 (50.0)	
Missing	2	1	0	1	
Presence of CLS (surgery)					0.261
CLS−	31 (75.6)	9 (90.0)	10 (83.3)	12 (63.2)	
CLS+	10 (24.4)	1 (10.0)	2 (16.7)	7 (36.8)	
Missing	15	3	8	4	
TAM (surgery)					0.062
Low	30 (73.2)	10 (100)	7 (58.3)	13 (68.4)	
High	11 (26.8)	0 (0.0)	5 (41.7)	6 (31.6)	
Missing	15	3	8	4	
Patients considered for CAAs analysis					0.163
No	23 (41.1)	7 (53.8)	10 (50.0)	6 (26.1)	
Yes	33 (58.9)	6 (46.2)	10 (50.0)	17 (73.9)	
Patients considered for distant adipocytes analysis					0.712
No	17 (30.4)	5 (38.5)	6 (30.0)	6 (26.1)	
Yes	39 (69.6)	8 (61.5)	14 (70.0)	17 (73.9)	

*P*-values are from the Fisher exact test and Kruskal-Wallis test when comparing categorical and continuous variables against 3 categories BMI, respectively. *BMI* body mass index, *CAAs* cancer-associated adipocytes, *CLS* crown-like structures, *ILC* invasive lobular carcinoma, *IQR* interquartile range, *NST* invasive carcinoma of no special type, *PEPI* preoperative endocrine prognostic index, *PgR* progesterone receptor, *SD* standard deviation, *TAM* tumor-associated macrophages.

**Fig. 1:**
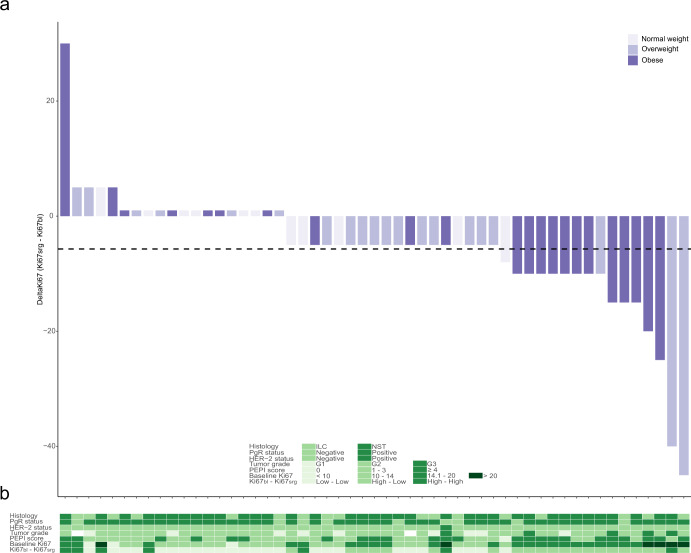
Patient Ki67 suppression and tumor characteristics. **a** Patient Ki67 suppression ordered from low to high Ki67 suppression, expressed in percentage, is defined as Ki67_srg_-Ki67_bl_. Each bar, representing a patient, is colored according to BMI category. The dashed line represents the arithmetic mean of Ki67 suppression. **b** Tumor characteristics by histology, baseline PgR status, baseline HER-2 status, baseline tumor grade, PEPI score, baseline Ki67 index, and “Ki67_bl_–Ki67_srg_” groups are indicated by colored boxes under each patient. BMI body mass index, HER-2 human epidermal growth factor receptor 2, Ki67_bl_ Ki67 at baseline, Ki67_srg_ Ki67 at surgery, ILC invasive lobular carcinoma, NST invasive carcinoma of no special type, PgR progesterone receptor status, PEPI preoperative endocrine prognostic index.

After 4 months of neoadjuvant letrozole, tumor Ki67 expression decreased in 34 patients, remained identical in 14 patients, and increased in 4 patients (Fig. 1). The mean pre-treatment Ki67 index was 15.9 (median: 15, interquartile range (IQR): 10–20). The mean post-treatment Ki67 index was 10.1 (median: 10, IQR: 5–10). The overall mean Ki67 suppression was −5.7 (median: -5, IQR: (-10)–0). According to BMI category, the mean Ki67 suppression was respectively: -2.1 (normal weight, median: 0, IQR: (-5)–0), -7.0 (overweight, median: -5, IQR: (-5)–0), -6.4 (obese, median: -10, IQR: (-10)–0).

### Association between BMI and anti-proliferative response

**Fig. 2:**
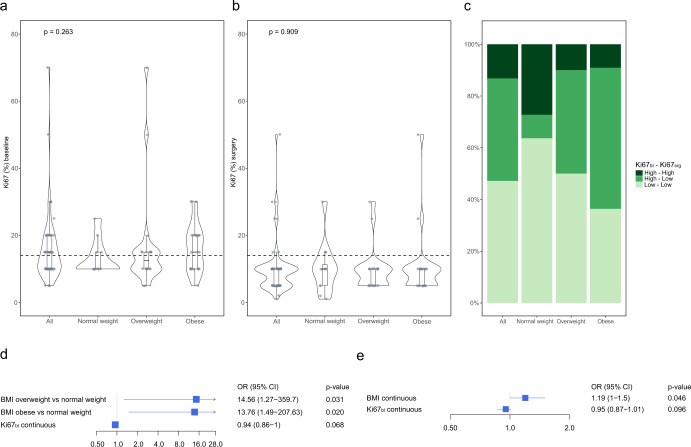
Association between Ki67 index and BMI. **a** Distribution of Ki67_bl_ according to BMI. Violin plots indicate the probability density of the data, and box plots represent the median (bold line) and interquartile range (rectangle). Dots report the distribution of the observed values. The dashed line represents the 14% Ki67 cut-off. **b** Distribution of Ki67_srg_ according to BMI. Violin plots indicate the probability density of the data, and box plots represent the median (bold line), interquartile range (rectangle) and the lower and upper whiskers (the two lines outside the box). Dots report the distribution of the observed values. **c** Stacked bar plot of percentage of Ki67_bl_–Ki67_srg_ groups according to BMI category. The Ki67_bl_–Ki67_srg_ groups are displayed on top of each other and are indicated by different colors. Low–low = Ki67_bl_ and Ki67_srg_ < 14%. High–low = Ki67_bl_ ≥ 14% and Ki67_srg_ < 14%. High–high = Ki67_bl_ and Ki67_srg_ ≥ 14%. Multivariable logistic models adjusted for continuous Ki67_bl_ considering BMI as categorical **(d)** or continuous **(e)** variable. All reported P-values are from Kruskal-Wallis and Wald tests. BMI body mass index, CI confidence interval, OR odds ratio, Ki67_bl_ Ki67 at baseline, Ki67_srg_ Ki67 at surgery.

No significant association was found between Ki67_bl_ and BMI (*p* = 0.263, Fig. 2a) or between Ki67_srg_ and BMI (*p* = 0.909, Fig. 2b). We evaluated the association between BMI and anti-proliferative response defined by the “Ki67_bl_–Ki67_srg_” groups and by Ki67_srg_ (residual Ki67). Figure 2c shows the distribution of Ki67_bl_–Ki67_srg_ groups according to patients’ BMI. We observed a significantly larger proportion of patients with obesity with “high Ki67_bl_–low Ki67_srg_” tumors (12 out of 22, 54.5%), as compared to patients with overweight (8 out of 20, 40.0%) and patients with normal weight (1 out of 11, 9.0%, *p* = 0.031). Then, we further explored the relationship between BMI and anti-proliferative response using regression models. We found a significant association between BMI (overweight vs normal weight and obese vs normal weight) and the anti-proliferative response according to the responders vs non responders categorization in the multivariable analysis adjusted for Ki67_bl_ (odds ratio (OR) obese vs normal weight: 13.76, 95% Confidence Interval (95%CI): 1.48–207.63, *p* = 0.020, Fig. 2d). A similar association was observed when considering BMI as a continuous variable (OR for 1 kg/m^2^ increase: 1.19, 95%CI: 1.00–1.50, *p* = 0.046, Fig. 2e). Of note, we did not find any evidence of a nonlinear effect for BMI. Similar effects, although not statistically significant, were observed for residual Ki67 and BMI as categorical variable (obese vs normal weight, OR: 3.02, 95%CI: 0.50–20.97, *p* = 0.223), and residual Ki67 and BMI as continuous variable, at the multivariable level (OR: 1.08, 95%CI: 0.95–1.29, *p* = 0.230).

### Association between adipocyte size, BMI and anti-proliferative response

**Fig. 3:**
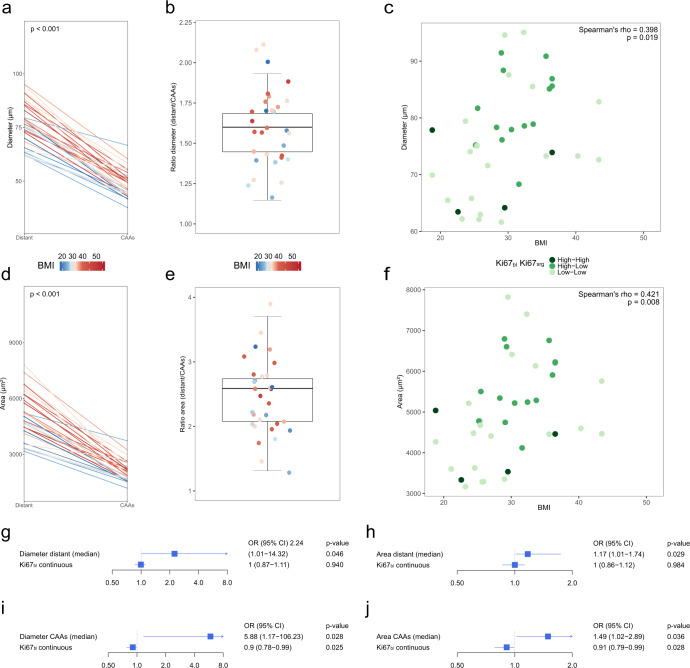
Adipocyte size and associations with BMI and anti-proliferative response. **a**, **d** Parallel plots show the variation of adipocyte diameter and area between distant and CAAs. **b**, **e** Box plots show the distribution of the adipocyte diameter and area ratio (distant / CAAs). Each line and each dot corresponds to a patient and is colored according to ascending BMI. Reported *P*-values are from paired Wilcoxon tests. Box plots represent the median (bold line), interquartile range (rectangle), and the lower and upper whiskers (the two lines outside the box). **c**, **f** Correlation between BMI and distant adipocytes diameter **(c)** and area **(d)**. Spearman’s rho and corresponding P-values are reported on the top-right corner of the plots. The color indicates the Ki67_bl_-Ki67_srg_ groups. Low–low = Ki67_bl_ and Ki67_srg_ < 14%. High–low = Ki67_bl_ ≥ 14% and Ki67_srg_ < 14%. High–high = Ki67_bl_ and Ki67_srg_ ≥ 14%. **g**–**j** Multivariable logistic models adjusted for continuous Ki67_bl_ investigating the association of adipocyte size and the anti-proliferative response: considering distant **(g**, **h)** or CAAs **i**, **j** and diameter **(g**, **i)** or area **(h**, **j)**. All reported *P*-values are from Wald tests. BMI body mass index, CAAs cancer-associated adipocytes, CI confidence interval, Ki67_bl_ Ki67 baseline, Ki67_srg_ Ki67 surgery, OR odds ratio.

First, we compared the median adipocyte area and the median adipocyte diameter of distant adipocytes and CAAs for each patient, and confirmed, in agreement with our previous work^22^, that CAAs were smaller than distant adipocytes (Fig. 3a, b). The mean diameter was 48.7 µm (median: 47.3, IQR: 44.1–52.5, standard deviation (SD): 6.2) for CAAs and 76.6 µm (median: 76.1, IQR: 70.7–83.9, SD: 9.4) for distant adipocytes (paired Wilcoxon test, *p* < 0.001). The mean area was 2086 µm^2^ (median: 1949, IQR: 1671–2343, SD: 551.3) for CAAs and 5003 µm^2^ (median: 4785, IQR: 4339–5833, SD: 1200) for distant adipocytes (paired Wilcoxon test, *p* < 0.001). Second, we investigated the correlation between BMI and adipocyte size. BMI as categorical variable (normal weight, overweight or obese) was significantly correlated with adipocyte size with regard to area (Kendall’s tau = 0.287, *p* = 0.041, and Kendall’s tau = 0.340, *p* = 0.007, CAAs and distant adipocytes respectively) and diameter (Kendall’s tau = 0.304, *p* = 0.031, Kendall’s tau = 0.323, *p* = 0.011, CAAs and distant adipocytes respectively). The correlation was further confirmed when considering BMI as continuous variable, both with regard to area of CAAs and distant adipocytes (Spearman’s rho = 0.355 *p* = 0.042; Spearman’s rho = 0.421 *p* = 0.007, respectively) and diameter of CAAs and distant adipocytes (Spearman’s rho = 0.352 *p* = 0.044; Spearman’s rho = 0.399 *p* = 0.011, respectively). Third, we explored the association between adipocyte size, BMI and anti-proliferative response (Fig. 3c). The “high Ki67_bl_–low Ki67_srg_” group had larger adipocytes as compared to the “low Ki67_bl_–low Ki67_srg_” group (*p* < 0.001, *p* = 0.012, CAAs and distant adipocytes respectively) and to the “high Ki67_bl_–high Ki67_srg_” group (*p* < 0.001, *p* = 0.007, respectively). Then, we assessed through multivariable regression analysis the relationship between adipocyte size and anti-proliferative response. We found that larger distant adipocyte size (diameter and area) is an independent predictor of anti-proliferative response (OR for 5 µm increase in diameter: 2.24, 95%CI: 1.01–14.32, *p* = 0.046, OR for 100 µm^2^ increase in area: 1.17, 95%CI: 1.01–1.74, *p* = 0.029, Fig. 3g, h). Similar associations were observed for CAAs (Fig. 3i, j). Considering residual Ki67, the direction of association terms were conserved but did not reach statistical significance (OR: 1.52, 95%CI: 0.87–3.19, *p* = 0.148, OR: 1.08, 95%CI: 0.98–1.23, *p* = 0.124, distant adipocyte diameter and area, respectively and OR: 1.35, 95%CI: 0.65–3.77, *p* = 0.458, OR: 1.06, 95%CI: 0.90–1.36, *p* = 0.514 CAAs diameter and area, respectively).

**Fig. 4:**
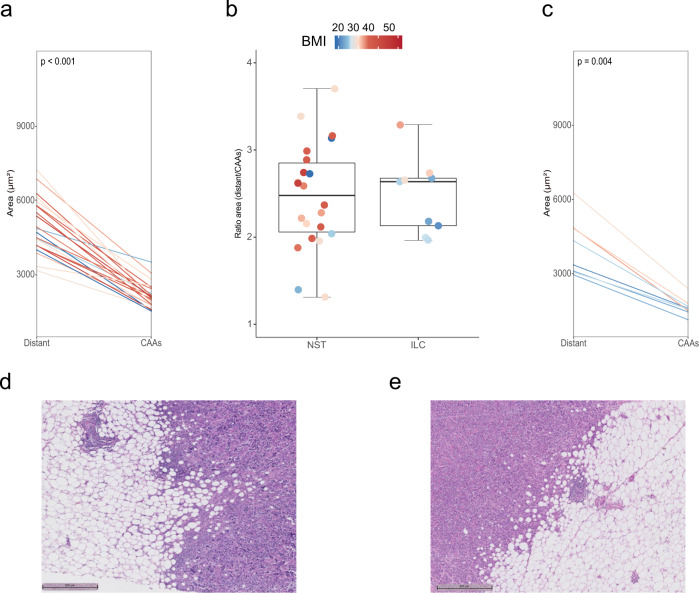
Adipocyte size and histological subtypes. **a**, **b** Parallel plots show the variation of adipocytes area between distant and CAAs (left panel, NST; right panel, ILC). Each line represents a patient and is colored by ascending BMI. **c** Box plots show the adipocyte area ratio (distant/CAAs). Each dot, representing a patient, is colored by ascending BMI. Box plots represent the median (bold line), interquartile range (rectangle), and the lower and upper whiskers (the two lines outside the box). **d**, **e** H&E slides showing the interaction between CAAs and cancer cells (left slide, NST; right slide, ILC). All reported *P*-values are from paired Wilcoxon tests. CAAs cancer-associated adipocytes, ILC invasive lobular carcinoma, NST invasive carcinoma of no special type.

### Association between adipocyte size, BMI, CLS, and TAM

We subsequently investigated the presence of CLS in the mammary adipose tissue. CLS were present in the adjacent mammary tissue of 10 out of the 41 patients which could be evaluated (24%; 7 obese, 2 overweight, and 1 normal weight). We found a positive linear correlation between CLS density and adipocyte size (Supplementary Fig. 2a, b), whereas no significant correlations were found with age (Spearman’s rho = 0.132, *p* = 0.411; all post-menopausal patients) and BMI (Spearman’s rho = 0.26, *p* = 0.100). The presence of CLS (CLS− vs CLS+) was also associated with a higher distant adipocyte diameter and area (Supplementary Fig. 2c, d). This association was confirmed by univariable and multivariable linear regression analysis (Supplementary Fig. 2e, f). In the latter, adjusted for continuous BMI, the direction of the estimate is maintained, although it did not reach statistical significance (Supplementary Fig. 2g, h).

Finally, we evaluated the association between TAM categorized in low TAM and high TAM, adipocyte size, and BMI. We showed a positive association, although not significant, between TAM and adipocyte size where increased adipocyte diameter and area were associated with higher presence of TAM (Wilcoxon: *p* = 0.055, *p* = 0.067, Supplementary Fig. 3a, b). The same trend was found at the univariable and multivariable level, adjusted for continuous BMI, but the association did not reach statistical significance (Supplementary Fig. 3c–f). Of note, no association was found between TAM and continuous BMI (IQR: 23.55–34.12, IQR: 29.20–34.65, *p* = 0.110, low TAM and high TAM respectively), while a positive association was observed between TAM and CLS density (IQR: 0–0, IQR: 0–0.1, *p* = 0.027, low TAM and high TAM, respectively), as well as between TAM and age (IQR: 60–71, IQR: 68.5–78, *p* = 0.026 low TAM and high TAM respectively).

### Adipocyte size and histological subtypes

We finally explored the association between adipocyte size and BC histology (NST vs ILC; *n* = 25, *n* = 10, respectively) and we observed slightly smaller adipocytes in ILC vs NST tumors for both CAAs and distant adipocytes (Fig. 4a–c). The mean diameter was 50.09 µm (median: 49.32, IQR: 45.93–53.28, SD: 6.31) and 45.73 µm (median: 44.40, IQR: 43.71–46.89, SD: 5.16) for NST and ILC CAAs, respectively (unpaired Wilcoxon test, *p* = 0.062); 78.54 µm (median: 77.17, IQR: 73.28–85.48, SD: 8.75) and 72.89 µm (median: 75.08, IQR: 63.45–78.58, SD: 9.88) for NST and ILC distant adipocytes, respectively (unpaired Wilcoxon test, *p* = 0.128). The mean area was 2207 µm^2^ (median: 2112, IQR: 1831–2450, SD: 562.26) and 1809 µm^2^ (median: 1681 IQR: 1608–1874, SD: 430.20) for NST and ILC CAAs, respectively (unpaired Wilcoxon test, *p* = 0.042); 5248 µm^2^ (median: 4921, IQR: 4462–6132, SD: 1137.30) and 4513 µm^2^ (median: 4676, IQR: 3335–5243, SD: 1215.56) for NST and ILC distant adipocytes, respectively (unpaired Wilcoxon test, *p* = 0.118). The interaction between adipocytes and cancer cells at the invasive front is displayed in Fig. 4d, e for NST and ILC respectively. We further explored this association through univariable and multivariable regression analysis. The direction of the estimates confirmed the aforementioned trend of ILC having smaller adipocytes as compared to NST tumors, but the association did not reach statistical significance (Supplementary Fig. 4).

## Discussion

The impact of BMI on ET has been evaluated retrospectively in a few adjuvant clinical trials hypothesizing a lower efficacy of ET in patients with overweight and patients with obesity, but not strongly supported by the evidence^23–25^. By contrast, in the neoadjuvant setting, a study consisted of 109 patients, suggested that high BMI might be a positive predictor of AI (exemestane) response based on tumor reduction rate, and another recent study found no difference in Ki67 changes and radiological response according to BMI^19,20^. A large neoadjuvant clinical trial evaluated ET response in post-menopausal ER positive BC patients, using changes in Ki67 before and after 2 weeks of neoadjuvant aromatase inhibition. In the aforementioned trial, four groups of patients were identified, after Ki67 dichotomization, based on Ki67 index at baseline and at 2 weeks. The authors found that a group of patients with the anti-proliferative response to neoadjuvant aromatase inhibition had a better prognosis as compared to the patients whose tumors had still a high Ki67 value at surgery^17^. However, it is unknown if obesity and/or mammary adiposity could influence ET response, measured as changes in Ki67. To address this question, we evaluated the predictive value of BMI and mammary adiposity on anti-proliferative response following 4 months of neoadjuvant aromatase inhibition with letrozole, in postmenopausal patients with ER positive BC. Furthermore, we explored the association between anti-proliferative response and BMI/adipocyte size, and BC histology.

First, we showed that patients with overweight and patients with obesity had a higher Ki67 suppression following neoadjuvant letrozole as compared to patients with normal weight. We also found that in the obese group there was a higher proportion of patients with high Ki67_bl_ and low Ki67_srg_ as compared to patients with overweight and patients with normal weight. In our cohort, higher BMI was associated with anti-proliferative response irrespective of Ki67 at baseline. Considering that BMI is inaccurate in describing the adiposity of specific fat depots, we also assessed mammary adipocyte size as alternative measure of mammary adiposity. In line with our previous work^22^, we showed a correlation between BMI and adipocyte size, although it is moderate. As for BMI, we found that adipocyte size (area and diameter) is a positive predictor of anti-proliferative response, showing that patients with larger distant adipocytes were more likely to have an anti-proliferative response. We also showed that patients with overweight and patients with obesity presented more frequently CLS in the mammary adipose tissue as compared to patients with normal weight, in line with the literature^26–28^. Furthermore, there exists a positive correlation between adipocyte size and CLS density confirming that patients with increased adiposity tend to have an inflammatory state, measured as CLS density, in the mammary adipose tissue^7,28^. We also detected a positive association between the presence of TAM and adipocyte size, but not with BMI category. It has been described that ET might contribute to deplete adipocyte progenitors and support the adipocyte hypertrophy only in women with high BMI leading to a higher risk of metabolic diseases. Therefore, increased adipocyte size might be also considered as an early indicator of adverse metabolic effects of endocrine therapies in a specific subgroup of patients^29^.

The association between BMI or adipocyte size on one hand and the anti-proliferative response in BC on the other hand is surprising to some extent. Several studies have consistently reported that postmenopausal patients with a high BMI and ER-positive BC had worse disease outcomes as compared to patients with a lower BMI^2^. We and others have shown that adipocyte size positively correlates with BMI^7,22,30^. This correlation is however not perfect as a subgroup of patients with low BMI also present CLS and larger mammary adipocytes^7^. In our study, we showed that patients with adverse prognostic characteristics such as high BMI, increased adipocyte size, and presence of CLS in the adjacent mammary tissue achieve more frequently an anti-proliferative response to neoadjuvant letrozole, a response which is considered as a favorable prognostic factor^17,31^. Several studies reported a higher aromatase activity in the presence of larger mammary adipocytes and macrophages infiltration^7,26^. We might therefore speculate that the mitogenic effect due to the estrogens, reflected by the Ki67 index, might be greater in those patients with larger adipocytes and higher aromatase activity as compared to patients with low BMI. Hence, the aromatase inhibition might lead to a greater suppression of the mitogenic effect reflected by the Ki67 reduction in those patients having high BMI and/or larger adipocytes. We acknowledge the complexity of the biological context located at the carrefour between increased adiposity, adipose tissue dysfunction, breast cancer and treatment response. Our study sheds light on part of the system but the biological meaning through which anti-proliferative response might be positively influenced by BMI and adipocyte size remains to be fully elucidated.

In this study, we also explored the size of mammary adipocytes in the two most prevalent histological subtypes of BC (ILC vs NST). A previous study described a different expression pattern of lipid metabolism proteins between these BC histological subtypes, showing a higher expression of certain lipases in ILC^32^. Hypothesizing that lobular cancer cells may have a stronger interaction with surrounding adipocytes due to looser cell-cell adhesion, we found that CAAs in patients with ILC seems to be smaller as compared to CAAs of patients with NST BC supporting the hypothesis of a higher delipidation in adipocytes from ILC. By contrast, at the histological level, ILC has been described having a fat-avoiding growth^33^. This observation has not been found in our cohort, but we cannot exclude a role of the letrozole in shaping the size of adipocytes and modulating the interaction between cancer cells and CAAs. These observations will need to be further investigated in future studies.

Patients and samples were selected based on the present interaction between cancer cells and adipocytes. This means that patients with denser breast parenchyma, in which the tumor has limited contact with the adipose tissue, are not considered in the analysis. Further studies are required to elucidate whether the extent of tumor-adipocyte interaction might influence the biology and anti-proliferative response of the tumor.

There are several limitations in this study that could be addressed in future research. So far, there is no standard definition of anti-proliferative response. We, therefore, used the definitions reported in recent neoadjuvant clinical trials^17,34^. However, one should be careful as various definitions of Ki67 suppression could induce small changes in categorizing patients as responders or non-responders. The number of patients was relatively small, therefore a clinical validation in larger cohorts of patients treated with neoadjuvant AIs is needed. Another aspect was the evaluation of adipocyte size in the pre-treatment biopsies that was only possible for 21 patients (37%) due to small quantity of AT presented on those samples and hence we excluded them from the analysis. Future studies should therefore possibly also consider taking pre-treatment biopsies in the adjacent mammary tissue to allow the evaluation of the adipocytes.

To conclude, our results suggest that letrozole might have a higher anti-proliferative efficacy in patients with increased adiposity. These observations, which need to be confirmed in larger series of patients, further highlight the need of investigating treatment efficacy and BC biology according to the adiposity of the patient.

## Methods

### Patients and slides

The study population was composed by 66 patients treated at the Institut Jules Bordet, Brussels, Belgium. They received four months of neoadjuvant ET with oral letrozole 2.5 mg once daily and underwent surgery between November 2004 and December 2014. Of note, no patients achieved a pathological complete response. The level of expression of ER and PgR was measured by immunohistochemistry using the Quick Score on tumor sample from tru-cut biopsy (pre-treatment) and from surgery specimens (post-treatment) and centrally reviewed as per study protocol (Supplementary Table 1).

Patients were eligible for the present sub-study if they met the following criteria: (1) a primary invasive carcinoma of NST or ILC, (2) postmenopausal status at diagnosis, (3) ER positive status of the tumor at diagnosis, (4) available information on BMI recorded at last on the day of oncological surgery. A total of 56 out of 66 patients met the inclusion criteria (Supplementary Fig. 1). BMI (kg/m^2^) was categorized according to the World Health Organization (WHO) criteria: normal weight (18.5–24.9 kg/m^2^), overweight (25–29.9 kg/m^2^), obese (≥30 kg/m^2^)^35^. Patients that were underweight (BMI < 18.5 kg/m^2^) were excluded given their potentially adverse prognosis^36^.

Multiple hematoxylin and eosin (H&E)-stained breast tissue slides, from surgery specimens, were available for adipocyte analysis. After a revision by an expert breast pathologist (D.L.), 112 H&E slides were chosen for subsequent analysis. We excluded the slides with damaged adipose tissue or low quality of the staining. H&E-stained slides were scanned using a Nanozoomer digital scanner (c10730-12, Hamamatsu) with a 40x objective (0.228 mm/pixel). Mammary adipocytes (distant from the tumor and CAAs) were selected and annotated in digital slides as follows: adipocytes distant from the tumor were defined as those being at least 2 mm away from cancer cells as well as 2 mm away from fibrosis area and epithelial structures. CAAs were defined as the three first lines of adipocytes at the invasive front of the tumor and as maximum 2 mm within the tumor starting from the invasive front^22^. A minimum of 500 individual distant adipocytes and 500 individual CAAs per patient were measured to determine median adipocyte area (µm²) and median adipocyte diameter (µm) using HALO^®^ software, version 2.3 (vacuole module, version 2.2 - Indica Labs, Corrales, NM, USA). Measurements with a diameter under 30 µm were not considered in our analysis in order to exclude artefacts. Immunohistochemistry for CD68 was performed on sections from formalin-fixed paraffin-embedded tumor blocks, using the monoclonal mouse anti-human CD68 antibody (clone KP1), ready to use (prediluted). An expert breast pathology (D.L.) counted and recorded the number of CLS in the adjacent mammary tissue. The CLS density (CLS/cm^2^) was calculated as the number of CLS per square centimeter of adipose tissue. On average 5 blocks per patient were stained for CD68. CD68-positive cells were further counted and recorded, as percentage of TAM in each scanned tumor slide. TAM were categorized into low or high category according to 10% cut-off.

The project has been approved by the ethics committee of the Institut Jules Bordet (CE2844, 3 May 2018) and all patients provided written informed consent.

### Ki67 assessment

Centralized Ki67 assessment was performed. Ki67 delta suppression was defined as the difference between Ki67 at surgery and at baseline (Ki67_srg_ – Ki67_bl_). The primary endpoint was to evaluate the anti-proliferative response. Comparing Ki67 levels at baseline with those at surgery after Ki67 dichotomization using a 14% cut-off, four groups of patients were identified: “low Ki67_bl_–low Ki67_srg_” (Ki67_bl_ and Ki67_srg_ < 14%); “high Ki67_bl_–low Ki67_srg_” (Ki67_bl_ ≥ 14%, Ki67_srg_ < 14%); “high Ki67_bl_–high Ki67_srg_” (Ki67_bl_ and Ki67_srg_ ≥ 14%); and “low Ki67_bl_–high Ki67_srg_” (Ki67_bl_ < 14%, Ki67_srg_ ≥ 14%). Responders were the patients in the “high Ki67_bl_–low Ki67_srg_” group, “high Ki67_bl_–high Ki67_srg_” were considered as non-responders while “low Ki67_bl_–low Ki67_srg_” were excluded from the analysis. No patients were classified into the “low Ki67_bl_–high Ki67_srg_” group. An alternative measure of response was used, and it was defined by the Ki67 index at surgery, also called residual Ki67. Responders and non-responders were identified as the patients with a residual Ki67 value of <14% or ≥ 14%, respectively. These definitions were chosen for consistency with previous neoadjuvant clinical trials. Three patients were excluded from the analysis because of missing Ki67 values.

The PEPI consisting of pathological tumor size, pathological node status, Ki67 index, and ER status of residual tumors after neoadjuvant ET, was assessed according to the algorithm of Ellis^16^. PEPI score was categorized into three risk groups as follows: low (0 points), moderate (1–3 points), and high (≥4 points). 1 out of the 56 patients (1.9%) had a PEPI score = 0, 29 out of 56 patients (53.7%) had a PEPI score between 1 and 3, and 24 out of 56 patients (44.4%) had PEPI score ≥ 4.

### Statistical Analysis

We evaluated the association between baseline clinico-pathological characteristics and BMI using Fisher exact test and Kruskal-Wallis test when comparing categorical and continuous variables against the three BMI categories. Associations between continuous and (ordinal) categorical variables were assessed using Wilcoxon and Mann-Kendall tests. Correlation between continuous BMI and continuous adipocyte measurements was assessed using the Spearman rank test. Univariable and multivariable Firth logistic regression models with anti-proliferative response or residual Ki67 as the response variables, BMI (continuous and categorical), adipocyte size (diameter and area) as the predicting variables of interest, and Ki67_bl_ as covariate in the anti-proliferative response, were used to assess the associations between BMI, adipocyte size, and anti-proliferative response or residual Ki67. In the continuous setting, non-linear effects were explored during the model-building phase resorting to regression penalized cubic splines. In a similar manner, we also performed multivariable linear regression analysis, adjusted for BMI, to evaluate the association of adipocyte size with CLS presence (CLS+ vs CLS−), TAM category (high vs low), and histological BC subtype (ILC vs NST). All odds ratios (OR) were computed for 1 kg/m^2^ increase in BMI but for 5 μm and 100 μm^2^ for adipocyte’s diameter and area respectively. Patients with missing values were excluded from the analysis. The Wilcoxon rank-sum test was performed to evaluate the association between adipocyte size and histology. The distribution of BMI in patients with adipocyte size data included in the multivariable analysis (*n* = 41) was not significantly different from the whole patient population (*n* = 56), nor from that in the excluded patients. P-values were 2-sided and considered as statistically significant at the conventional level of .05. Consistently, 95% confidence intervals (CIs) were appropriately computed with the functions provided in the logistf, and rms packages. Statistical analyses were performed using R version 4.0.2^37^.

### Reporting summary

Further information on research design is available in the Nature Research Reporting Summary linked to this article.

## Supplementary information

Supplementary data 1

## Data Availability

The data that support the findings of this study are available upon request to the corresponding author after signature of a Data Access Agreement. The data are not publicly available due to the personal nature of the containing information.

## Code availability

The code and a simulated dataset, mimicking the actual data, are available online on Code Ocean repository^38^: https://codeocean.com/capsule/1861469/tree (10.24433/CO.7285076.v1).
