# Supplementary material for: The association between adiposity and anti-proliferative response to neoadjuvant endocrine therapy with letrozole in post-menopausal patients with estrogen receptor positive breast cancer
Source: NPJ Breast Cancer. 2022 Aug 4;8:90. doi: 10.1038/s41523-022-00453-7 (PMC9352750; doi:10.1038/s41523-022-00453-7)
Supplement: Supplementary file 1 — Supplementary data 1 [file 41523_2022_453_MOESM1_ESM.pdf]

Supplementary Information to Isnaldi et. al. “The association between adiposity and anti-proliferative response to neoadjuvant endocrine therapy with letrozole in post-menopausal patients with estrogen receptor positive breast cancer”

Table of Contents

***Supplementary Table and Figures* 2**

Supplementary Table 1: Distribution of ER and PgR expression at baseline and at surgery. 3

Supplementary Figure 1: Flow diagram for patient enrollment and sample availability. 4

Supplementary Figure 2: Association between adipocytes size and CLS adjusted for BMI........... 5

Supplementary Figure 3: Association between adipocytes size and TAM adjusted for BMI......... 6

Supplementary Figure 4: Association between adipocytes size and histology adjusted for BMI... 7

Supplementary Table and Figures

	All	Normal weight	Overweight	Obese	p-value
QuickScore of ER (baseline)					0.276
3	1	0 (0.0)	0 (0.0)	1 (4.5)	
5	1	1 (7.7)	0 (0.0)	0 (0.0)	
6	1	0 (0.0)	1 (5.0)	1 (4.5)	
7	4	0 (0.0)	4 (20.0)	1 (4.5)	
8	46	12 (92.3)	15 (75.0)	19 (86.4)	
Missing	1	0	0	1	
QuickScore of ER (surgery)					0.259
0	2	1 (7.7)	0 (0.0)	1 (4.3)	
5	2	2 (15.4)	0 (0.0)	0 (0.0)	
6	2	0 (0.0)	1 (5.0)	1 (4.3)	
7	6	1 (7.7)	1 (5.0)	4 (17.4)	
8	44	9 (69.2)	18 (90.0)	17 (73.9)	
QuickScore of PgR (baseline)					0.865
0	5	2 (16.7)	2 (11.1)	1 (5.6)	
3	1	0 (0.0)	0 (0.0)	1 (5.6)	
4	4	2 (16.7)	0 (0.0)	2 (11.1)	
5	1	0 (0.0)	1 (5.5)	0 (0.0)	
6	4	1 (8.3)	1 (5.5)	2 (11.1)	
7	9	1 (8.3)	5 (27.9)	3 (16.7)	
8	24	6 (50.0)	9 (50)	9 (50.0)	
Missing	8	1	2	5	
QuickScore of PgR (surgery)					0.813
0	26	6 (46.2)	10 (50.0)	10 (45.5)	
2	2	1 (7.7)	0 (0.0)	1 (4.5)	
3	4	1 (7.7)	2 (10.0)	1 (4.5)	
4	5	1 (7.7)	1 (5.0)	3 (13.6)	
5	3	2 (15.4)	1 (5.0)	0 (0.0)	
6	4	0 (0.0)	2 (10.0)	2 (9.1)	
7	5	2 (15.4)	1 (5.0)	2 (9.1)	
8	6	0 (0.0)	3 (15.0)	3 (13.6)	
Missing	1	0	0	1	

Supplementary Table 1: Distribution of ER and PgR expression at baseline and at surgery.
ER, estrogen receptor; PgR, progesterone receptor.

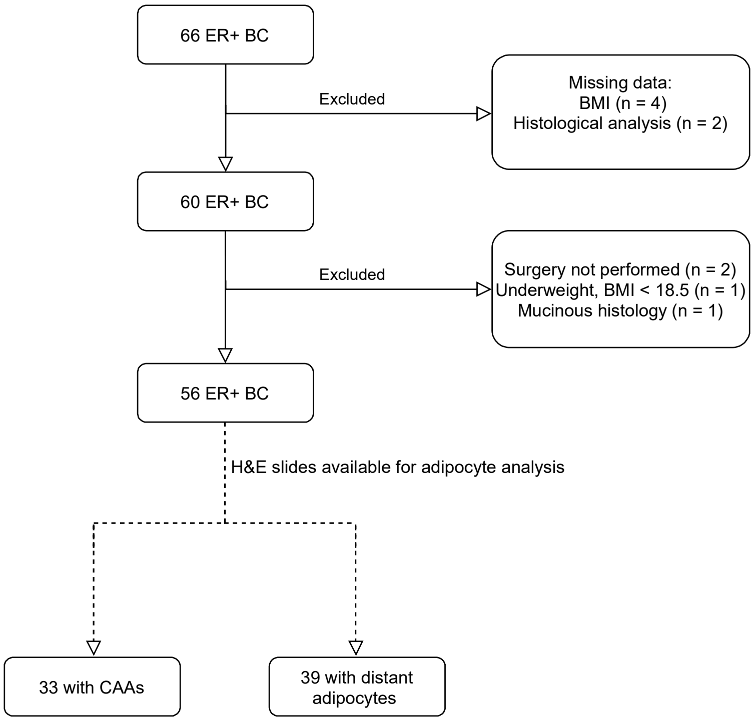

Supplementary Figure 1: Flow diagram for patient enrollment and sample availability.

BC, breast cancer; BMI, body mass index, CAAs, cancer-associated adipocytes; ER+, estrogen receptor positive; H&E, hematoxylin and eosin.

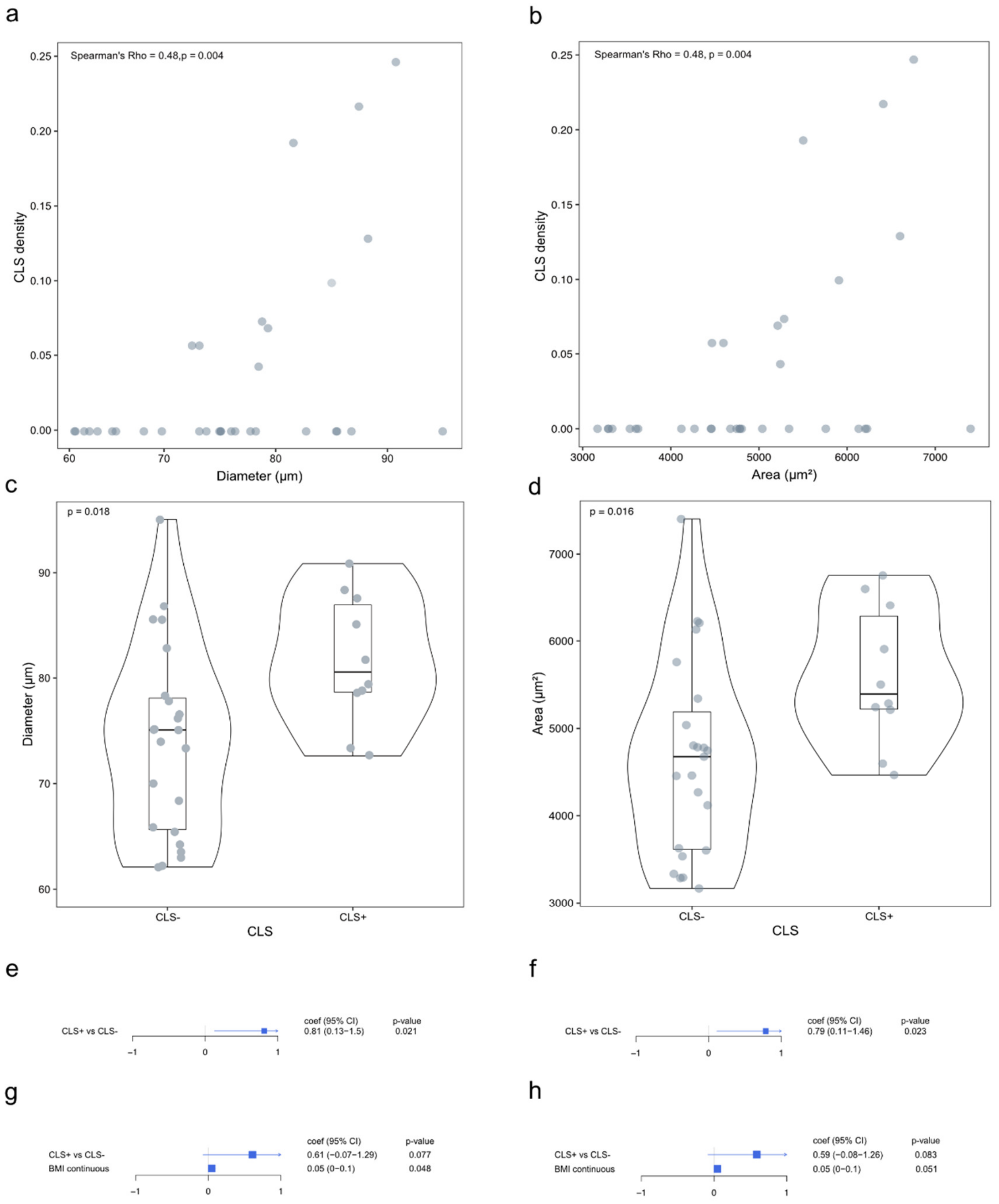

Supplementary Figure 2: Association between adipocytes size and CLS adjusted for BMI.

(a,b) Correlation between distant adipocyte diameter (a) and distant adipocyte area (b) and CLS density. Spearman's rho and corresponding P values are reported on the top-left corner of the plots.

(c,d) Distribution of distant adipocyte diameter (c) and distant adipocyte area (d) according to the presence of CLS (CLS- vs CLS+). Violin plots indicate the probability density of the data, and box plots represent the median (bold line) and interquartile range (rectangle). Dots report the distribution of the observed values.

(e,g) Diameter of distant adipocytes. (f,h) Area of distant adipocytes. (e,f) Univariable models. (g,h) Multivariable models. Adipocyte sizes are scaled prior to compute the general linear models.

BMI, body mass index; CI, confidence interval; CLS, crown-like structures; coef, coefficient.

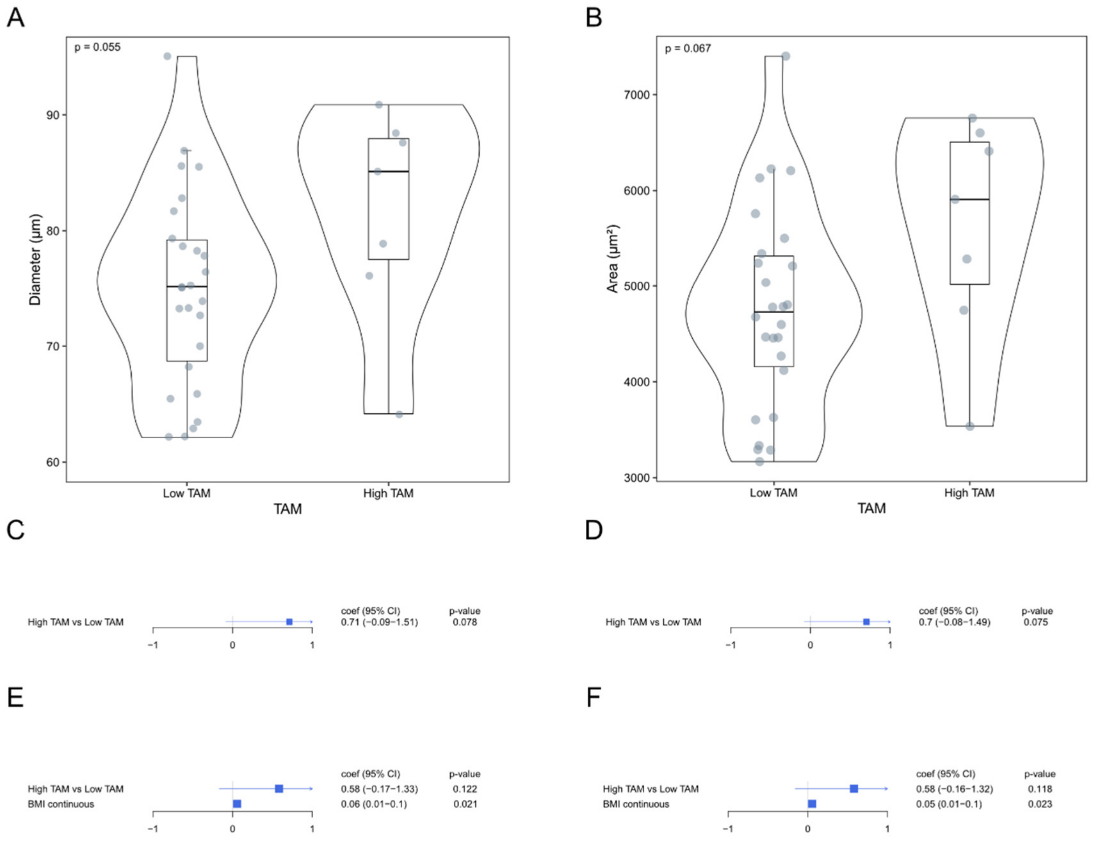

Supplementary Figure 3: Association between adipocytes size and TAM adjusted for BMI.

(a,b) Distribution of distant adipocyte diameter (a) and distant adipocyte area (b) according to the presence of TAM (Low TAM vs High TAM). Violin plots indicate the probability density of the data, and box plots represent the median (bold line) and interquartile range (rectangle). Dots report the distribution of the observed values.

(c,e) Diameter of distant adipocytes. (d,f) Area of distant adipocytes. (c,d) Univariable models. (e,f) Multivariable models. Adipocyte sizes are scaled prior to compute the general linear models.

BMI, body mass index; CI, confidence interval; coef, coefficient; TAM, tumor-associated macrophages.

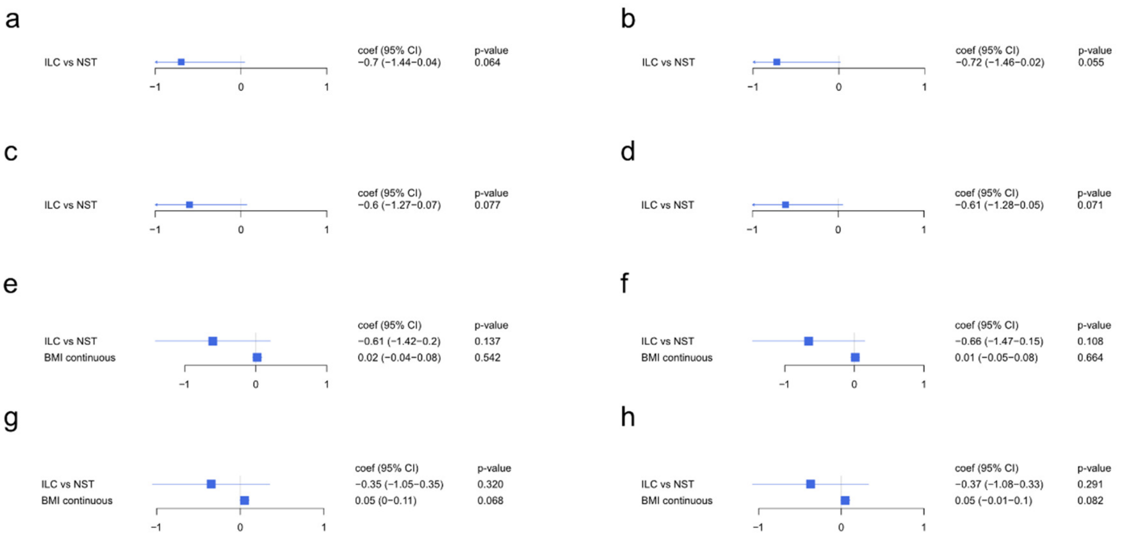

Supplementary Figure 4: Association between adipocytes size and histology adjusted for BMI.

(a,c,e,g) Diameter of CAAs and distant adipocytes respectively. (b,d,f,h) Area of CAAs and distant adipocytes respectively. (a,b,c,d) Univariable models. (e,f,g,h) Multivariable models. Adipocyte sizes are scaled prior to compute the general linear models.

BMI, body mass index; CAAs, cancer-associated adipocytes; CI, confidence interval; coef, coefficient; ILC, invasive lobular carcinoma; NST, invasive carcinoma of no special type.
